# IDbSV: An Open-Access Repository for Monitoring SARS-CoV-2 Variations and Evolution

**DOI:** 10.3389/fmed.2021.765249

**Published:** 2021-12-13

**Authors:** Abdelmounim Essabbar, Souad Kartti, Tarek Alouane, Mohammed Hakmi, Lahcen Belyamani, Azeddine Ibrahimi

**Affiliations:** ^1^Medical Biotechnology Laboratory (MedBiotech), Bioinova Research Center, Rabat Medical and Pharmacy School, Mohammed Vth University, Rabat, Morocco; ^2^Emergency Department, Military Hospital Mohammed V, Rabat Medical & Pharmacy School, Mohammed Vth University, Rabat, Morocco

**Keywords:** SARS-CoV-2, COVID-19, genomic variations, database, mutation

## Abstract

Ending COVID-19 pandemic requires a collaborative understanding of SARS-CoV-2 and COVID-19 mechanisms. Yet, the evolving nature of coronaviruses results in a continuous emergence of new variants of the virus. Central to this is the need for a continuous monitoring system able to detect potentially harmful variants of the virus in real-time. In this manuscript, we present the International Database of SARS-CoV-2 Variations (IDbSV), the result of ongoing efforts in curating, analyzing, and sharing comprehensive interpretation of SARS-CoV-2's genetic variations and variants. Through user-friendly interactive data visualizations, we aim to provide a novel surveillance tool to the scientific and public health communities. The database is regularly updated with new records through a 4-step workflow (1—Quality control of curated sequences, 2—Call of variations, 3—Functional annotation, and 4—Metadata association). To the best of our knowledge, IDbSV provides access to the largest repository of SARS-CoV-2 variations and the largest analysis of SARS-CoV-2 genomes with over 60 thousand annotated variations curated from the 1,808,613 genomes alongside their functional annotations, first known appearance, and associated genetic lineages, enabling a robust interpretation tool for SARS-CoV-2 variations to help understanding SARS-CoV-2 dynamics across the world.

## Introduction

The Coronavirus Disease 2019 outbreak (COVID-19) caused by the Severe Acute Respiratory Syndrome Coronavirus 2 (SARS-CoV-2), has spread from Wuhan China in November 2019 to over 214 countries and territories around the world causing more than 4 million deaths (as August 2021) (1). Concerted efforts have been made in sequencing, analyzing and sharing SARS-CoV-2 genomes all around the world to control the spread of the virus and in particular to assess the virulence of the variants in circulation (2). In the absence of evidence of mutational escape from the currently developed treatments, one should continuously track all possible variations (3, 4). Monitoring SARS-CoV-2's variation dynamics is critical for the treatment of COVID-19 and ensuring the effectiveness of potential vaccines plays a central role in reinforcing international efforts to control the spread of viruses. So far, several databases have been published focusing on the genetic variants of SARS-CoV-2. GISAID is a pathogenic virus database that provides options to search for SARS-CoV-2 sequences based on their location and date of collection alongside an analytical tool for sequence alignment and visualization (5). The abundance of sequencing data on GISAID and other databases such as NCBI Genbank (6), ViPR allowed the development of more specific tools for monitoring SARS-CoV-2 evolution (7). Pangolin (Phylogenetic Assignment of Named Global Outbreak Lineages) was developed to help the assignment of likeness between SARS-CoV-2 genomes according to a dynamic lineage nomenclature scheme (8). However, Pangolin were dedicated to the classification of SARS-CoV-2 by clades, which have been determined on the basis of several variants of current genetic markers instead of a systematic analysis of all individual variations. In the same context, after over a year of COVID-19, several tools have been developed including Nextstrain (9), BioAider (10), Coronapp (11), CoV-Seq (12), ViruSurf (13), NGDC (14), CoV-GLUE (15), Favicov (16), and IDP 2.0 (17) to provide analysis of SARS-CoV-2 sequences. Yet most of these tools either settle for the annotation of an input given sequence, or lack information associated with the genetic variations such as functional interpretation, location and date of appearance, and associated lineages which are essential for exploring the time course and potential routes of transmission of SARS-CoV-2. Likewise, GESS (18) provide information about single nucleotide variants (SNVs) within a chosen genomic region or protein, or in a certain country/area of interest, however, it misses information about the other types of variations (INDELS and MNV) that played a crucial role in the evolution of SARS-CoV-2 and enhancing its spreading capacities (19). The International Database of SARS-CoV-2 variations (IDbSV) was developed to close these gaps. IDbSV is an open repository, with monthly scheduled updates, hosting curated data about SARS-CoV-2 genetic variations identified from the analysis of high-quality SARS-CoV-2 genome sequences. In the next sections, we present a brief overview of the main genomic findings, with special focus on the most dominant variations, their first appearance and associated lineages well as the main functions implemented within IDbSV.

## Materials and Methods

### Data Collection

Complete nucleotide sequences of SARS-CoV-2 genomes were collected from the GISAID EpiCov^TM^ (https://www.epicov.org/epi3/), belonging to 188 territories and distributed over five continents as follows: Africa (1.57%), Asia (8.62%), Europe (63.81%), North America (21.23%), Oceania (1.86%) and South America (2.91%) and the date of samples collection was between December 24 2019 to July 28th, 2021. (The list of genomes used to build the current version of IDbSV can be found as Supplementary Table).

### Quality Control

Only high-quality complete genomes with available metadata were considered for the variations analysis. Genomes were first filtered considering genomes completeness (>29,000 bp), coverage (<1%) and percentage of undefined bases (<5% Ns). The remaining sequences were selected according to the availability of their geographical and temporal metadata.

### Variants Calling and Functional Annotation

**Figure 1:**
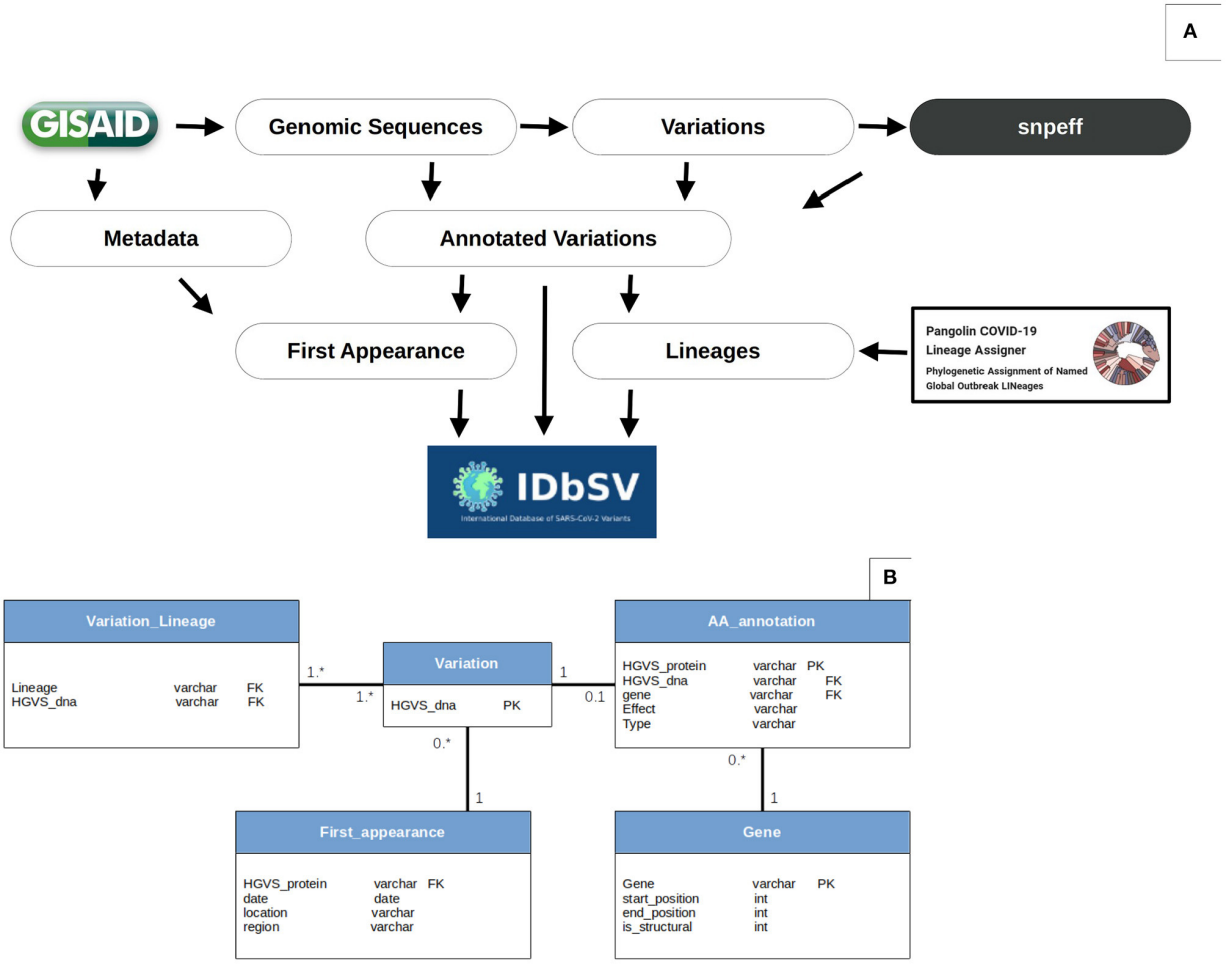
Data analysis workflow and database schema. **(A)** Variations' extraction workflow: the flowchart resumes the procedure of variation extraction and annotation. The variations are identified using MINIMAP2, SAMTOOLS and BCFTOOLS and annotated using snpEff. These SNPs are then associated with appropriate strain's metadata and lineages according to GISAID (www.epicov.org/epi3/) and PANGOLIN (pangolin.cog-uk.io). The data is then exported from CSV files to relational tables as SQL files. Finally, the outputs are deployed online on a monthly basis. Data processing scripts are available openly in https://github.com/mouneem/IDbSV and the extracted list of strains can be found in Supplementary Table 1. **(B)** Database Schema. Using this object-oriented architecture, instead of the standard VCF table, allowed further queries and alleviation of search queries flexibility and reduced storage.

High-quality sequences were mapped individually against the SARS-CoV-2 reference genome Wuhan-Hu-1/2019 (Genbank ID: NC_045512.2) using Minimap2-2.17 (20) to identify variants. The resulting SAM files were sorted and converted to BAM formats before calling the genetic variants in Variant Call Format (VCF) using multiple-sample pileup (mpileup) from the SAMtools suite (21). Variation's functional significance was predicted using snpEff 5.0e (22) based on each variant's relative location and nucleic acid alteration (Figure 1A). The variants identified in more than 1% of studied samples were considered as recurrent variations and the variations identified in more than 10% of the studied samples were considered as hotspot variations. The identified DNA variations and Amino acid mutations were represented in the HGVS standards and nomenclature to enable systematic exploration of our database via semantic web tools and APIs (23).

### Metadata Annotation

The identified list of variants was first linked to their appropriate strain's contextual information according to GISAID geographical and temporal metadata. Then, based on their amino acid annotations, each variant was associated with appropriate lineages according to Rambaut's nomenclature proposal for SARS-CoV-2 lineages (pangolin.cog-uk.io/) (8).

### Platform Architectural Design and Structure

Data processing and analysis were conducted using Python-3.8 and R-3.6, and the web platform was implemented using PHP 7 and a relational database connection.

An object-oriented architecture was designed and implemented in a relational database (MySQL) to store the annotated variants instead of the conventional spreadsheet file (CSV/VCF) to allow further flexibility when formulating search queries and alleviate database load by reducing data duplication. The database architecture and relationships between tables is shown in Figure 1B. The Human Genome Variation Society (HGVS) nomenclature (23) were used as primary keys for both nucleotide and amino acids variations to join tables.

## Results

### Distribution of Variations

**Figure 2:**
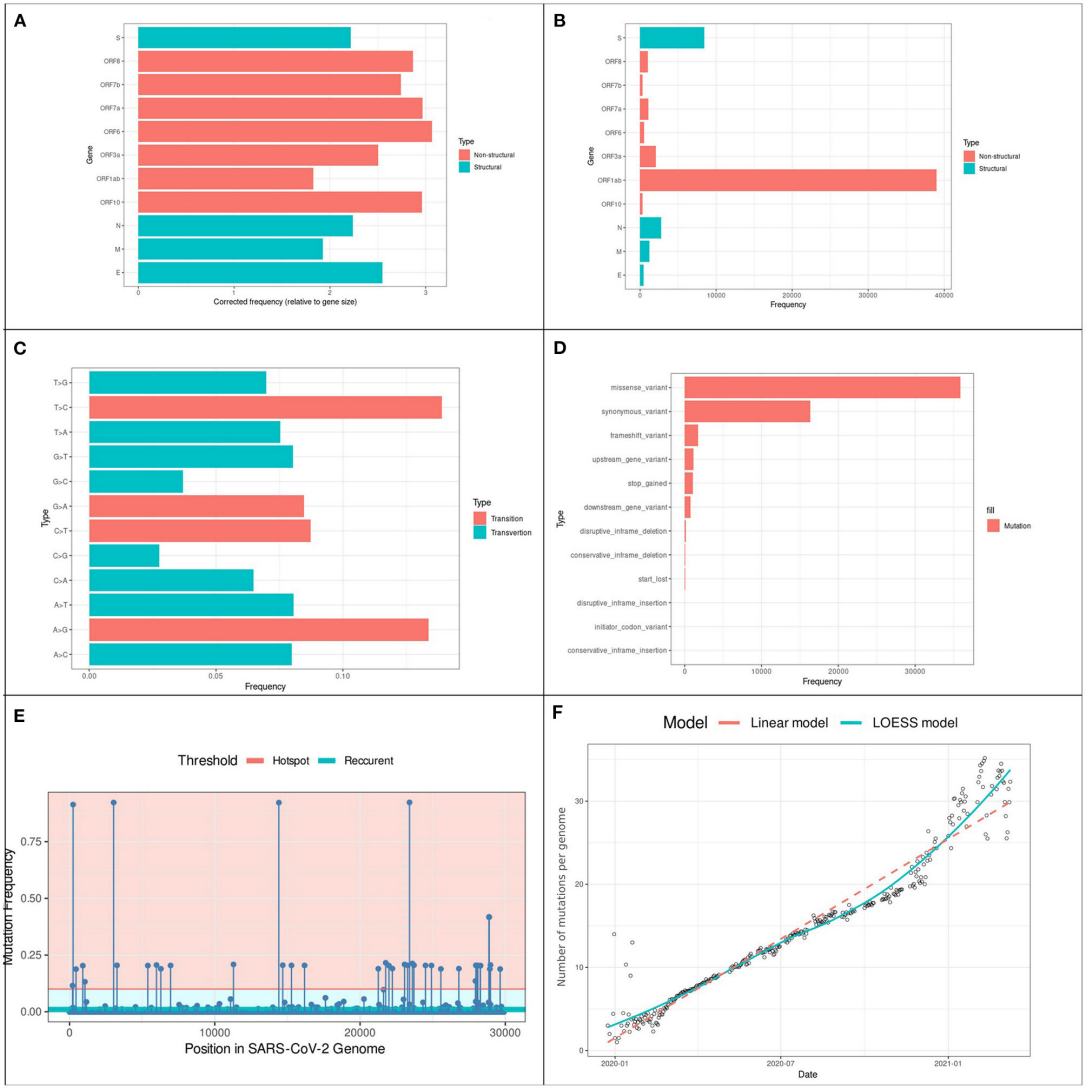
Prevalence and distribution of types of variants in 201,951 SARS-CoV-2 genomes **(A)** variants locations: the y-axis represents the gene location of variants, and the x-axis the rate of each gene. **(B)** Gene length corrected frequency **(C)** variants type frequencies: the y-axis represents the type of variants, and the x-axis the rate of each variation. **(D)** Types frequency: The prevalence of each type of variation: the y-axis represents the type of variant, and the x-axis the rate of each type. **(E)** Distribution of the 36,967 variants across the SARS-CoV-2 genome. The Lollipop plot illustrates the location of variations. The horizontal lines represent the threshold for recurrent (light-blue) and hotspot (Red) variations. All types of variations are included (non-synonymous, synonymous, and intergenic). Forty two (hotspot) variations occurred in more than 10% of analyzed genomes from which 241C > T, 3037C > T, 14408C > T and 23403A > G were identified in more than 91% (N ≧ 184525). **(F)** Variations accumulative on SARS-CoV-2 genomes over 16 months. Points on the scatter-plot represent genomes, the x-axis represents the collection date and y-axis is the number of variations. Linear (red) and Local Polynomial (light-blue) regression models are plotted to visualize the trend of evolution over the past 16 months of the pandemic.

From over 2,683,000 genomic sequences available on the GISAID database on the 5th of August 2021, we selected 1,808,613 (67.8%) complete high-quality SARS-CoV-2 genomic sequences. Our analysis of these sequences revealed the presence of 60,148 distinct variations coding for 57,581 different amino acid mutations across the 11 SARS-CoV-2 genes. The accumulation of variations, especially in structural regions gives viruses a selective advantage for host invasion and adaptation, higher translatability of more virulent strains, and drug resistance (24, 25). Figure 2 shows the different types of variants identified, their positions, and their frequencies. We identified 27.2% of the variants in regions coding for structural proteins including spike (S): 14.8%, nucleocapsid (N): 4.8%, membrane (M): 2.1%, and envelope (E): 0.8%. while the remaining 72.2% were distributed over six Open-Reading Frame genes (ORF3a, ORF6, ORF7a, ORF7b, ORF8, and ORF10) as it is shown in Figures 2A,E. From an evolutionary perspective, the rate of variations can also be a key parameter to assess the speed of viral evolution. We found an evolution rate of 18.86 variations per genome on average and we estimated the increase in the cumulative count of variation by ~0.08 [std error 0.001] additional variant each day as it is shown in Figure 2F. In addition, among the 60 thousand variations, 98.2% were located in coding regions of the genome and distributed as follows: 67.2% missense variations and 30.8% synonymous variations, resulting in a 2.18 Non-synonymous/Synonymous variations ratio.

### Frequency of Variations

Despite the low rate of recurrent variations, some variations were widely spread worldwide. Figure 2E shows the distribution of variations and their frequencies: 162 variations were identified with a frequency >1% while only 40 variations were identified as hotspot variations (frequency > 10% of the total samples). Expectedly, the two missense mutations 23403A > G and 14408C > T were identified in nearly 1.6 million genomic samples (93.8%), this mutation was linked to the B.1 lineages that spread from Europe to become the most prevalent form of the virus around the world. The frequency of the remaining variations changed according to geographic location as described in Table 1. Other noteworthy lineages that spread to over 10% of the population are B.1.1, B.1.1.7 and B.1.617.2 which correspond to the current literature (3, 26).

**Table 1:** Top-20 recurrent variants and their frequencies in 5 different geographic regions.

**SNP**	**Africa**	**Americas**	**Asia**	**Europe**	**Oceania**
23403A > G	94.51%	90.63%	71.26%	94.38%	88.87%
3037C > T	94.40%	90.48%	71.81%	94.22%	88.90%
14408C > T	92.51%	90.50%	71.68%	94.11%	88.84%
241C > T	93.73%	89.48%	71.76%	93.84%	80.69%
28881G > A	49.86%	15.30%	39.72%	33.51%	74.85%
28883G > C	49.81%	15.19%	39.42%	33.42%	74.83%
28882G > A	49.81%	15.19%	39.48%	33.44%	74.82%
1163A > T	0.00%	0.03%	3.24%	0.17%	69.03%
18555C > T	0.00%	0.56%	0.30%	0.37%	68.34%
16647G > T	0.00%	0.09%	0.07%	0.08%	68.00%
23401G > A	0.00%	0.00%	0.00%	0.02%	67.98%
7540T > C	0.00%	0.00%	0.00%	0.00%	67.98%
22992G > A	1.39%	0.62%	0.32%	7.73%	66.40%
25563G > T	15.20%	59.33%	22.60%	16.34%	8.51%
1059C > T	8.26%	54.38%	5.92%	6.35%	6.34%
27964C > T	0.00%	22.74%	0.55%	0.42%	1.05%
22227C > T	0.94%	0.31%	0.46%	20.24%	0.46%
6286C > T	0.67%	0.16%	0.42%	20.21%	0.32%
28932C > T	1.44%	0.11%	0.37%	20.04%	0.28%
445T > C	0.67%	0.09%	0.36%	19.97%	0.29%
29645G > T	0.72%	0.12%	0.51%	19.82%	0.28%

### Content and Features

**Figure 3:**
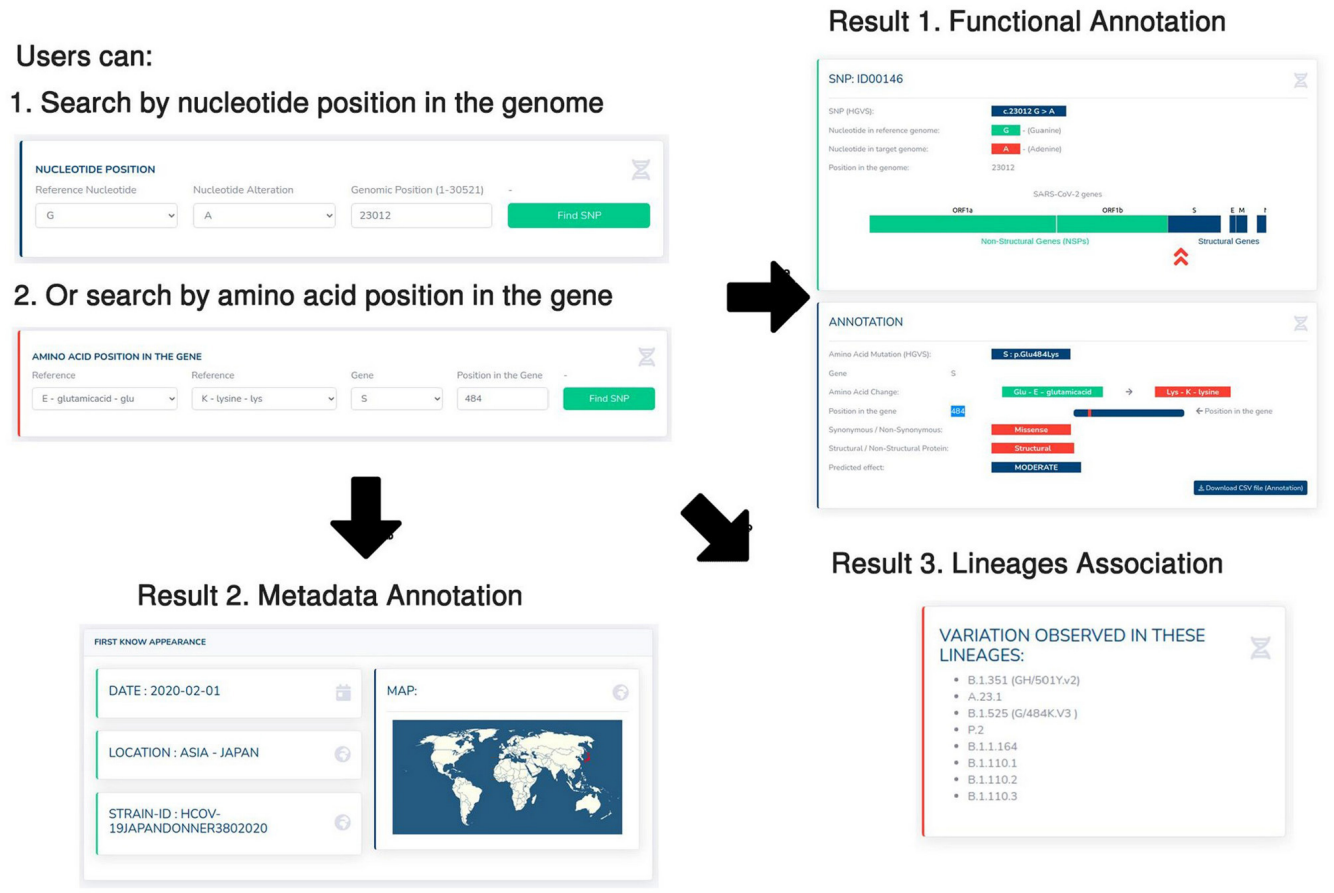
Case study of the 23012G > A variant. Screenshots from IDbSV web-portal providing contextual information about IDbSV c.23012G > A coding for the variation E484K in spike glycoprotein region. The users can start by searching for a genetic variation using its position in the genome (1), or its amino acid position in a specific gene (2). The database automatically returns three main results: (1) the functional annotation of the selected mutation (Position in the genome, associated amino acid mutation, type of mutation, predicted effect), (2) geographic and time information about the first known appearance of the selection variation, (3) the associated lineages with the selected variation.

The present findings in the previous sections were summarized in the interactive web-platform (accessible through http://IDbSV.medbiotech-lab.ma) to assist the navigation over thousands of annotated records via a user-friendly graphical user interface. A querying tool has been implemented in the platform to simplify the genome browsing by positions in genome and genes which allows the investigation of the variations occurring in specific regions or genes (Figure 3). Users may retrieve information about a specific variation by its position in the genome (Figure 3), by its position in a specific gene (Figure 3) or by the summarize table visible in the home page. User is automatically redirected to the page with functional annotation of the selected variation such as HGVS nomenclature, resulting Amino Acid mutation, position in the specific gene, type of mutation and the predicted impact of the mutation. For example, the screenshots in Figure 3 provide a demonstration of the database functionalities using the substitution of Guanine (G) by Adenine (A) in the 23,012 positions of SARS-CoV-2 genome which led to a missense change of glutamic acid (E) by Lysine (K) in the position 484 of the Spike protein (S: p.Glu484Lys).

Furthermore, the annotated list variations were linked to metadata information about the countries and regions of origin and date of collection. Which allows the users of IDbSV to extract the contextual information about the first known appearance of each variant (Figure 3). Moreover, we identified variations to their appropriate lineages following (8) nomenclature to allow further understanding of the global spread patterns and determinants. As shown in the show case example in (Figure 3), according to the IDbSV 23012G > A was first identified in Japan on 1st February 2020 (GISAID ID: HCOV-19JAPANDONNER3802020), then subsequently spread around the world across more than 8 lineages including the lineages of interest B.1.351, A.23.1 and B.1.525. This example not only showcases the use of the present database, but also highlights the importance of metadata such as appearance location and date and lineages association in the context of following the spread of SARS-CoV-2 and understanding the dynamics of the virus.

## Discussion

The global collaborative efforts have been, with no doubt, the key weapon in the fight against COVID-19. The massive efforts in sequencing and sharing SARS-CoV-2 genomes allowed investigators to reveal many previously unknown characteristics of COVID-19 in its diagnosis and treatment (27). IDbSV joins international efforts by providing comprehensive datasets on the genetic evolution of SARS-CoV-2 in time and space. The current version of IDbSV provides access to the result of analysis of over 1.8 million high quality complete genomes. The inclusion of a maximum number of genomic samples increases the statistical significance of our findings and allows the consideration of more recent variations and less pathogenic ones, which may raise more concerns in the future (28). In addition to the thousands of identified variations that can be browsed through the platform's GUI, the database provides access to their associated metadata. Furthermore, unlike the existing tools developed to assist monitoring SARS-CoV-2's evolution, IDbSV does not require any input file and/or computational knowledge to be used. Moreover, IDbSV can be used as an online annotation tool for the interpretation of mutations. These annotated variations are openly accessible using the GUI or API requests which enable the use of IDbSV for the development of other specific pipelines.

Until 5th August 2021, IDbSV hosted over 60 thousand variations extracted from the analysis of over 1.8 million SARS-CoV-2 genomes. It is interesting to note that the analysis of these strains revealed consistent results with the finding of more specialized studies (29–35). Yet we revealed a median variation rate of 18.6 variations per genome with an increasing rate of one more variation every 12.5 days, which is expectedly higher than what identified in earlier studies (36, 37). Noteworthy, 27.8% of variations were identified in regions coding for structural proteins, this put more emphasis on the importance of monitoring SARS-CoV-2 variations especially in these regions, as these structural proteins are the main targets of the currently developed vaccines) (38, 39).

## Summary

Since November 2020, IDbSV provided a complete atlas of SARS-CoV-2 genetic changes, with particular emphasis on recurrent and potentially harmful mutations. To the best of our knowledge, the current version of IDbSV (August 2021) provides open access to the largest repository of SARS-CoV-2 variations, with 60.148 annotated genetic changes curated from 1.8 million selected samples representing different regions and countries. Given the importance of monitoring the changes in virus transmissibility and severity, the goal of IDbSV is to provide an open-access and user-friendly platform for researchers and the public to browse SARS-CoV-2 variations in real-time. In addition to the functional annotation of the identified variations, IDbSV provides detailed information about the date of appearance, location of appearance and associated phylogenetic lineage of each variation. The results of these work produced an overview of circulating variations that provide guidance for public health measures to fight the pandemic. We plan to continuously update the platform monthly with new data and features as the fight against COVID-19 continues, to help researchers reveal and interpret new variations and potentially aid in drug and vaccine design.

## Data Availability Statement

The datasets presented in this study can be found in online repositories. The names of the repository/repositories and accession number(s) can be found in the article/Supplementary Material.

## Author Contributions

AE, SK, TA, MH, LB, and AI contributed to the analysis of genomic data and redaction of this manuscript. All authors have read and agreed to the published version of the manuscript.

## Funding

This research was funded by the Moroccan Ministry of Higher Education and Scientific Research (COVID-19 Program) and the Institute of Cancer Research (IRC).

## Conflict of Interest

The authors declare that the research was conducted in the absence of any commercial or financial relationships that could be construed as a potential conflict of interest.

## Publisher's Note

All claims expressed in this article are solely those of the authors and do not necessarily represent those of their affiliated organizations, or those of the publisher, the editors and the reviewers. Any product that may be evaluated in this article, or claim that may be made by its manufacturer, is not guaranteed or endorsed by the publisher.
